# Data and replication supplement for double auction markets with snipers

**DOI:** 10.1016/j.dib.2019.104729

**Published:** 2019-10-30

**Authors:** Paul Brewer, Anmol Ratan

**Affiliations:** a Economic and Financial Technology Consulting LLC, United States; b Department of Economics, Monash Business School, Monash University, Australia

**Keywords:** Markets, Double auction, Competitive equilibrium, Efficiency, Inequality, Numerical experiments, Simulations

## Abstract

We provide a dataset for our research article “Profitability, Efficiency and Inequality in Double Auction Markets with Snipers” [1]. This dataset [2] includes configuration files, raw output data, and replications of calculated metrics for our robot-populated market simulations. The raw data is subdivided into a hierarchy of folders corresponding to simulation treatment variables, in a 2 × 2 × 21 design for 84 treatments in total. Treatments variables include: (i) robot population ordering, either “primary” or “reverse”; (ii) two market schedules of agent's values and costs: equal-expected-profit “market 1” and unequal-expected-profit “market 2”; (iii) 21 robot populations identified by the number of Sniper Bots (0–20) on each side of the market. Each treatment directory contains a simulator input file and outputs for 10,000 periods of market data. The outputs include all acceptable buy and sell orders, all trades, profits for each agent, and market metrics such as efficiency-of-allocation, Gini coefficient, and price statistics. An additional public copy in Google Cloud is available for database query by users of Google BigQuery.

The market simulator software is a private product created by Paul Brewer at Economic and Financial Technology Consulting LLC. Free open source modules are available for tech-savvy users at GitHub, NPM, and Docker Hub repositories and are sufficient to repeat the simulations. An easier-to-use paid market simulation product will eventually be available online from Econ1.Net. We provide instructions for repeating individual simulations using the free open source simulator and the free container tool Docker.

**Table:** Specifications Table

Subject	Economics, Econometrics and Finance (General)
Specific subject area	Microeconomics
Type of data	Table Other: simulator configuration input in JSON (JavaScript Object Notation)
How data were acquired	Raw data: proprietary market simulator located at https://econ1.net The proprietary simulator uses Google Cloud to run batches of multiple independent simulations in parallel. Each individual simulation uses the free open source market simulator stored in the GitHub repository: https://github.com/drpaulbrewer/single-market-robot-simulator version 4.3.0 (commit 6c808424 …) Replicated Metrics data: calculations on raw data using Google BigQuery and provided SQL statements or provided scripts
Data format	Raw Analyzed
Parameters for data collection	Only buy and sell orders that met the market improvement rule were kept for this dataset. Discarded orders did not affect market.
Description of data collection	Relevant robot event data and then-existing market conditions are collected at these events: robot submits buy or sell order, trade occurs between robots, end of market period (profits, price statistics, Efficiency, Gini Coefficient).
Data source location	Economic and Financial Technology Consulting LLC, USA
Data accessibility	Brewer, Paul; Ratan, Anmol (2019), “Data and Replication Supplement for Double Auction Markets with Snipers”, Mendeley Data, V1 Direct URL to data: https://doi.org/10.17632/p9v66fzfhw.1
Related research article	Paul Brewer and Anmol Ratan Profitability, Efficiency, and Inequality in Double Auction Markets with Snipers Journal of Economic Behaviour and Organization https://doi.org/10.1016/j.jebo.2019.06.017

**Table:** 

**Value of the Data** • This dataset provides ready examples of simulated market event data (buy and sell orders, market trades, statistics such as high and low prices, Gini Coefficient, and Efficiency) for future comparison to other research involving robot trading, market processes, or inequality. • A subset of Economists and Computer Scientists interested in simulation or experimental approaches to robot trading, market processes, or fairness/inequality can benefit from these data when considering new simulations or analyses. Researchers in some areas of finance, market regulation, machine learning or artificial intelligence may also find the dataset of interest. • New analyses that reuse the data are possible. Topics that can be explored include: the determinants of low or high market efficiency, the determinants of low or high Gini coefficients of inequality, price variations and dynamics, the matching of buyers and sellers for trading induced by randomness and the market rules, the conditions for sniper strategies to be successful, and the properties of alternative measures of inequality. • To facilitate reproducibility and new research investigations, we provide configuration input data for the simulator and a detailed procedure for running a simulation. The input files are human and machine readable. They can be used to recreate similar simulations, or carefully altered to change the simulations. Changeable parameters include robots' value and cost parameters (thereby setting market demand and supply), the ZI robot and SB robot populations or use of other pre-programmed robots, and the relative speeds at which the robots arrive at the market. • The dataset expands upon the associated research article [1] by also providing raw data for additional cases that are useful to consider but were omitted from the main discussion because of space and scope limitations. Compared to the original analysis reported in Ref. [1], data here is extended with additional columns for sellers' data as well as buyers' data, and all tables regarding the effects of sniper population now contain additional rows for sniper proportions 0%–95% in 5% increments (instead of 0%, 5%, then 10% increments for 10–90%, and 95%). • The dataset includes both raw data from the simulations reported in Ref. [1] and a separate replication of the analysis (calculated data) using Google BigQuery, where a copy of the raw and calculated data exist. This copy enables online querying, filtering, and analysing of the raw and calculated data and can shorten the time to new insights.

## 1 Data

The *Data and Replication Supplement for Double Auction Markets with Snipers Dataset* [2] is a public Mendeley dataset that contains primary and supplemental raw data and a replication of analyzed data for the associated research article *Profitability, Efficiency, and Inequality in Double Auction Markets with Snipers* [1]. The raw data consists of the input configurations and market outputs (bids, asks, trades, profits, statistics) of robot trading under 84 different treatments. The replication of analyzed data consists of a re-creation of table and figure data similar to those in Ref. [1], including additional supplemental cases. SQL (Structured Query Language) and other scripts for transforming the raw data into the analyzed data are provided.

**Fig. 1:**
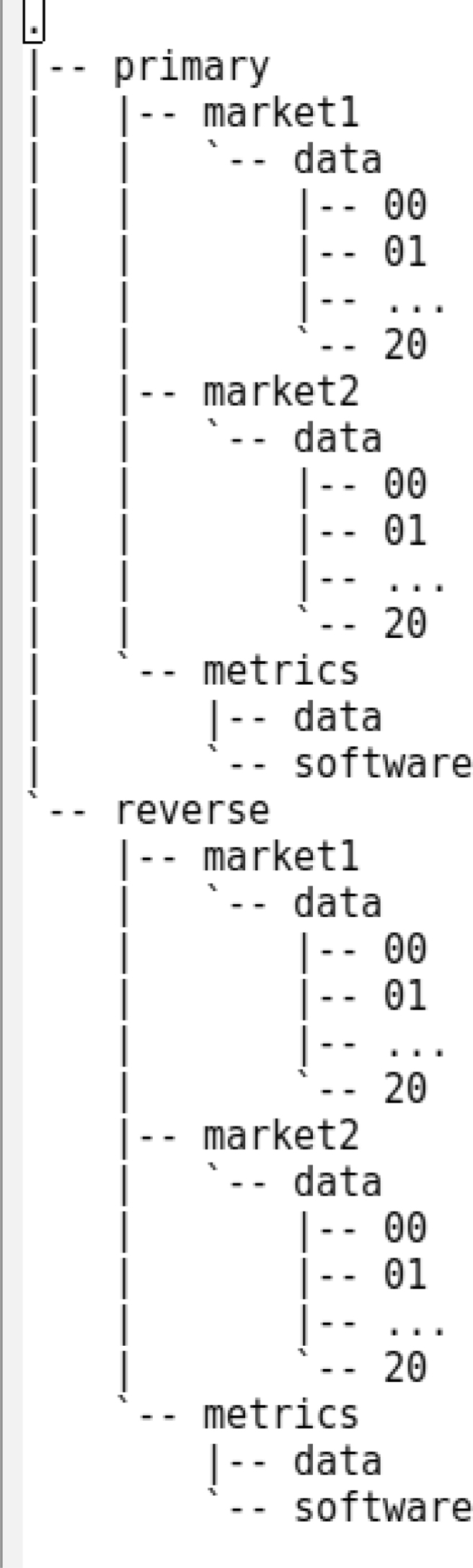
Summary of Dataset Directory Structure, without individual files.
• reverse.zip; a 1GB zip archive file. Unzipped, it yields 4GB of data files within the /reverse folder shown in Fig. 1. This folder will contain the raw and analyzed data for the market simulation treatments involving the “reverse” direction of robot replacement.

The Mendeley dataset contains three files:

• Archive-File-Guide.pdf; a metadata Adobe PDF document containing the definitions of columns for each.csv file format for the various file types, the properties in the sim.json file, and a quirks/errata page.
• primary.zip; a 1GB zip archive file. Unzipped, it yields 4GB of data files within the /primary folder shown in Fig. 1. This folder will contain the raw and analyzed data for the market simulation treatments involving the “primary” direction of robot replacement.

These folders correspond to the 2 × 2 × 21 treatment research design described in the methodology section below. Each treatment's raw data is in the numbered folder (00, 01, …, 20) at the bottom of the folder tree and contains a simulator input file (sim.json) and market data output files (buyorder.csv, sellorder.csv, trade.csv, ohlc.csv, profit.csv, and effalloc.csv).

The raw data for each treatment was generated by the single-market-robot-simulator open source module. This module is a portion of a simulator system available from Paul Brewer's company, Economic and Financial Technology Consulting LLC. This module is available from a public GitHub [3], NPM [4] or DockerHub [5] repository. The relevant version generating the /primary/* data is v4.3.0. Instructions for replicating a treatment are provided in the methodology section below.

Data files are organized as follows:

1. Data associated with each simulation treatment is organized in its own folder and includes the treatment variables as subfolder names in the order shown in these examples:
  **Input example:/primary/market1/data/12/sim.json**
  Here, this simulator configuration file belongs to a specific simulation involving the three treatment settings “primary”, “market 1”, and “case id 12” (60% SB robots, 40% ZI robots) in our 2 × 2 × 21 research design. The proportion of SB robots is always 5% multiplied by the folder number or case id. The “sim.json” file authoritatively defines the various agent and market parameters fed to the software for generating the accompanying output data. It can be used to repeat a treatment (subject to randomness).
  **Output example:/primary/market1/data/12/buyorder.csv**
  Again, this raw data output file belongs to the specific simulation involving the three treatment settings “primary”, “market 1”, and “case id 12”. As described in the “Archive-File-Guide”, files named “buyorder.csv” contain one buy order per row, for the 10,000 periods of data generated for that specific treatment.
2. Analyzed data (called “metrics”) similar to the data in tables and figures in Ref. [1] can be found in **/primary/metrics/data** and **/reverse/metrics/data.** All metrics involve a replicated analysis generated through Google BigQuery SQL and/or the gini-csv tool [7] from the original raw data and generally should not be expected to exactly match the original Tables and Figures in Ref. [1] either in professional polish, scope or numerically (e.g., compressed column names, more detailed scope, rounding issues in the least significant digit). Note that only the first treatment variable (primary vs. reverse) is used in the file path. The market, case id, or percentage of snipers are typically among the table columns reported in individual analysis files.
  Example:
  **/primary/metrics/data/Table2.csv** contains a table reporting an aggregate stats calculation like that reported in Ref. [1] at Table 2, p.493. The corresponding Google BigQuery SQL code that created this metrics data from raw data is given in **/primary/metrics/software/Table2.sql.**
3. The configuration file associated with a group of simulations executed via the proprietary Econ1.Net orchestration tools is in the associated **data** folder. The configuration files associated with individual treatments are produced from this file and are in the numbered treatment folder.
  Example:
  **/primary/market1/data/config.json** contains the proprietary Econ1.Net configuration file used to generate the various single-treatment configurations in **/primary/market1/data/NN/sim.json (**where NN = 00,01,02, …,20) for the open source portion of the simulator software.
4. Final Timestamps in Universal Time Zone (UTC) for the multi-simulation outputs are recorded in the relevant /*/**market1** or /*/ **market2** folder in a file called **original-file-name.** These timestamps are also the original zip filenames created by the Econ1.Net software running on Google Cloud.

### 1.1 Google BigQuery locations for data

A copy of archive data is stored in Google BigQuery database for ease of future analysis or replication. Therein, datasets and tables have global identifiers *‘project:dataset’*, *‘project:dataset.table’* or ‘*project*.*dataset*.*table*’, depending on context. These identifiers are as follows:

*Project* is always ‘eaftc-free’.

*Dataset* is one of:

‘BrewerRatan2019Market1’ or ‘BrewerRatan2019Market2’ for primary raw market data
‘BrewerRatan2019Market1R’ or ‘BrewerRatan2019Market2R’ for reverse raw market data
‘BrewerRatan2019Metrics’ for analyzed metrics based on primary data
‘BrewerRatan2019MetricsR’ for analyzed metrics based on reverse data

*Table* is one of:

‘buyorder’, ‘sellorder’, ‘trade’, ‘ohlc’, or ‘effalloc’ for primary or reverse raw market data;
or, generally, the associated archive file name without “.csv” or dashes for the analyzed data

The cases 00 to 20 contained in multiple files spread across the numbered data folders in the data archive are consolidated into single tables in Google BigQuery and are distinguished using the ‘caseid' column.

## 2 Experimental design, materials, and methods

### 2.1 Design

The 2 × 2 × 21 design involves every combination of the following three treatment variables:

• (2 choices) A sort order for replacing ZI robots with SB robots, either “primary” (buyer 1 and seller 1 (id 21) are first to change from ZI to SB) or “reverse” (buyer 20 and seller 20 (id 40) are the first to change from ZI to SB).
• (2 choices) A set of market value and cost parameters for each agent, either “market1” (theoretically equal profit for each robot agent at competitive equilibrium) or “market2” (theoretically unequal profit for each robot agent at competitive equilibrium). See Ref. [1], p.488 for the specific formulas.
• (21 choices) A population treatment of ZI and SB robots. Treatment N, 0≤N ≤ 20, has N SB Buyer robots, (20-N) ZI Buyer robots, N SB Seller robots, and (20-N) ZI Seller robots. See Ref. [1], section 3.2, p.490.

### 2.2 Methodology

#### 2.2.1 Original data generation and analysis procedures

Both authors participated in the process of data generation for exploration and topic identification with earlier prototypes of the simulator software.

##### 2.2.1.1 Original data generation procedure

For this dataset, Paul Brewer performed the data generation procedure four times, once for /primary/market1, /primary/market2, /reverse/market1, and /reverse/market2. The files reported here are the original files as generated and organized by the simulator, except that the files of buy and sell orders not meeting the bid/ask improvement rule (rejecbuyorder.csv and rejectsellorder.csv) have been omitted for reasons of space and relevance. The data generation procedure involves 2 steps:

1. Design a 21-simulation configuration in Econ 1.Net's web-based editor, creating the **config.json** file provided. The robot-replacement direction (primary or reverse) and the market value/cost parameters (market1 or market2) are held constant in this configuration and the variation from one simulation to another occurs in the robot populations from all ZI (0% SB) to all SB (100% SB). The robot replacement direction requirement (primary or reverse) determines the fields ‘morph.buyerAgentType’ and ‘morph.sellerAgentType’ (both “left” for primary; both “right” for reverse). These fields control transforming the first configuration (where fields ‘buyerAgentType’ and ‘sellerAgentType’ each contain an array of 20 “ZIAgent”) provided in config.json into the second configuration (where fields ‘buyerAgentType’ and ‘sellerAgentType’ each contain an array of 20 ‘KaplanSniperAgent’). The market1 or market2 value and cost parameters appear in the fields ‘buyerValues’ and ‘sellerCosts’ within the ‘common’ subsection as they are constant.
2. Run the **config.json** file in Econ1.Net for 10,000 periods on Google Cloud. Econ1.Net rents on demand a pre-emptible Google Compute Engine instance of type n1-highcpu-16 (16 CPUs, 14.4 GB RAM, 100 GB disk), and transfers the config.json file. The instance boots Linux, runs proprietary software, and the open source simulator from npm: single-market-robot-simulator (version 4.3.0 for “primary”, a somewhat later version for “reverse”). The proprietary software creates a primary folder and 21 simulation folders numbered 00 to 20. It creates the 21 **sim.json** files for the individual simulations, and orchestrates running independent copies of single-market-robot-simulator in 21 parallel processes, reporting progress and eventually delivering all raw market data output files within a single timestamped.zip file to a Google Drive folder.

Original Analysis Procedure.

1. Paul Brewer shares the relevant data folders in Google Drive as produced by the simulator with Anmol Ratan in Australia. Both researchers download the zip files, decompress the contents, and modify the data folder organization to suit tasks at hand.
2. Ad hoc analysis occurs with various tools. Procedures and numerical outputs are discussed and compared between the researchers during Google Hangout videoconferences.
3. The Gini coefficients - based on total profit aggregated over 10,000 periods - were calculated in Google Sheets in a custom function [6].
4. The final tables and figures were polished and finalized by Anmol Ratan in Stata.

In the research article [1], we focus on the “primary” treatments, and occasionally mention the “reverse” treatments in notes.

#### 2.2.2 Replicated analysis procedure for this archive

**Fig. 2:**
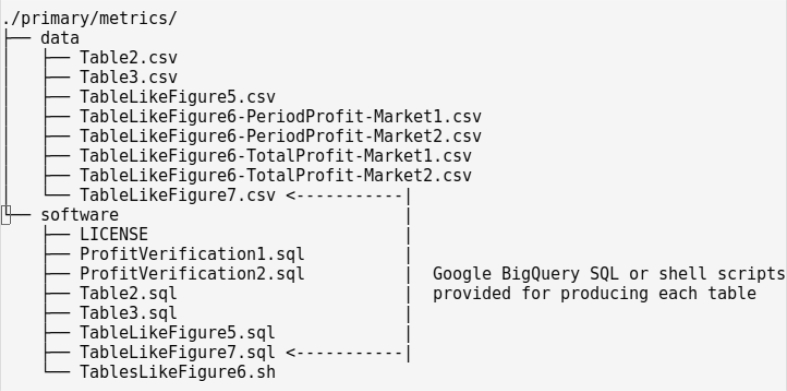
Folder listing of primary metrics files.

Note: The.sql queries can be executed directly against the data by anyone with access to Google's BigQuery web console [8] (free usage allowance, signup with Google Cloud required).

Fig. 2 provides a snapshot of the **data** and **software** subfolders for /primary/metrics/*. Each file in a **data** folder has been generated either by Google BigQuery or the gini-csv tool [7] for Gini Coefficients. The **software** folder provides relevant sql queries and scripts for producing the data files.

1. All raw data were first uploaded to Google's BigQuery database by consolidating files with the same name (e.g. buyorder.csv) and creating publicly accessible datasets corresponding to the first two treatment variables within the “eaftc-free” namespace:
  ○ “Primary” raw data is loaded into datasets “BrewerRatan2019Market1” and “BrewerRatan2019Market2″
  ○ “Reverse” raw data is loaded into datasets “BrewerRatan2019Market1R” and “BrewerRatan2019Market2R”.
2. Table names within each dataset are determined automatically by keeping the first part of each.csv file name: buyorder, effalloc, ohlc, sellorder, and trade.
3. The provided.sql queries in **software** were provided to generate SQL views (virtual tables, dependent on the raw data) and organized in these additional Google BigQuery datasets:
  ○ Analyzed “Primary” data is stored in BrewerRatan2019Metrics
  ○ Analyzed “Reverse” data is stored in BrewerRatan2019MetricsR

4. The data were downloaded from Google BigQuery into the files shown in Fig. 2.

#### 2.2.3 Quality control procedures for software and simulation data

##### 2.2.3.1 Procedures to test software and improve quality during software development

This describes the behavioural testing adopted by Paul Brewer and his company, Economic and Financial Technology Consulting LLC, when developing the mathematical and procedural open source simulator modules. The list below describes policies put in place and is not a list of steps. These policies arose naturally in the software industry, but are not universally adopted, and do not guarantee that bugs will not occur. Nothing can do so.

1. JavaScript software modules critical to operating the simulation are posted as open source on GitHub and NPM repositories. Specialization and delegation occur through this modular design.
  a. The simulator imports other modules listed below, reads the input configuration sim.json, and generates data for a single treatment.
  b. The double auction market trading takes place through the npm modules ‘market-example-contingent’, ‘market-engine’, and ‘market-pricing’.
  c. The robot agent strategies, profit accounting, and robot orchestration (including the notion of a period, a pool of buyers or sellers, and distributing a list of unit costs or unit values among such pools) occur in the npm module ‘market-agents’.
  d. Deciding what to log and when occurs in single-market-robot-simulator but the logging itself is delegated to the npm module ‘simple-isomorphic-logger’.
  e. Other functionality takes place as referenced in each module's package.json file.
2. Separation of these tasks into different modules allows for independent testing. Each module includes numerous software tests that exercise important functionality through a sequence of examples. These tests are located in the “test” subdirectory of each module.
3. Modules are designed to run both on a server (with nodejs) or on a researcher's PC in their web browser from the same lines of code, also known as *isomorphic JavaScript.* This reduces bugs and differences that might occur from mismatched code. A simulation running in Google Cloud should produce the same mathematical operations seen on the web browser. The cloud can run multiple simulations in parallel and for more periods or otherwise at larger scale.
4. Because these modules are open source, they qualify for free automated testing by the testing service Travis-CI. Reports from Travis-CI are available by clicking on the “build” badge in each module repository's documentation.
5. When a change to the software is posted to the GitHub repository, Travis-CI receives a notification, runs the automated testing, changes the “build” badge to passing/green or failing/red, and sends a test report to the software author (drpaulbrewer@eaftc.com). This aids in detecting unforeseen issues when restructuring code or adding new features.
6. When a bug or undesired operation is encountered, additional tests can be created that better define correct or desired behaviour or output.
7. Each change in a module that is published on NPM has a different NPM version identifier, such as 4.3.0 or 5.6.0, and overwriting old versions is not permitted. This is useful for uniquely identifying code for various quality and provenance related tasks. NPM also has a tool, npm, that is used to link one module to another, using these version identifiers to specify which version or sequence of versions is acceptable.

##### 2.2.3.2 Procedure to recover unit value and unit cost parameters from submitted orders

This procedure is a list of explicit steps and was performed as part of checking that the software performed as expected in reading the market parameters, particularly the robot traders value and cost parameters, and including them in relevant output in buy order and sell order raw data.

1. Each time a buyer robot submits a buy order (which may or may not be accepted by a seller) meeting the market improvement rule, a row is recorded in the simulator raw output data in file buyorder.csv. These rows are also combined into the buyorder tables in Google BigQuery. The relevant columns for recovering the original unit value parameters are ‘id’ and ‘buyerValue’. To scan and collate all of buy orders, periods, and treatments, a single query is sufficient.
2. For buyer values/primary/market1, execute in Google BigQuery console [8]:
  SELECT DISTINCT id,buyerValue
  FROM ‘eaftc-free.BrewerRatan2019Market1.buyorder'
  ORDER BY id asc, buyerValue desc;
3. Confirm that all values (there should be at most 3 per buyer agent) agree with the buyer unit values in the research design and no extraneous unit values are reported.
4. Repeat for market2, substituting “Market2” for “Market1” in step 2 above.
5. Repeat steps 2–4 for the reverse data, substituting Market1R or Market 2R
6. For seller costs, execute in Google BigQuery console:
  SELECT DISTINCT id,sellerCost
  FROM ‘eaftc-free.BrewerRatan2019Market1.sellorder'
  ORDER BY id asc, sellerCost asc;
7. Confirm that all costs (there should be at most 3 per seller agent) agree with the seller unit costs in the research design and no extraneous unit costs are reported. Transform the id number reported here to the “Seller Id” by subtracting 20. For example, the seller id number for the first seller is 21 in the software (id numbers 1–20 are buyers, 21–40 are sellers). The first seller is described as “Seller 1” in the research design.
8. Repeat steps 6–7 for Market2, Market1R, Market2R

#### 2.2.4 Replication procedure for an individual simulation treatment

This section describes installing and using simulation software from the internet on Windows, using Docker Desktop. While we expect the download archives to be well maintained, there are always security risks that you can mitigate by asking for local technical assistance.

For research transparency and provenance, it is desirable for other researchers to have the capability to replicate simulations with a tool similar to or part of the original software. Ideally, a version of the tool should be frozen and remain fixed over time and be independent of the latest changes and improvements. It should be convenient to run on commonly available computers, i.e. Windows, without overly complex installation. Ideally, the tool should be free – with source code available - and not involve permission from or tracking by the original researchers. Although source code should be available for techs to review, only “average researcher” knowledge should be required to run the tool to replicate a simulation.

These requirements are satisfied by a container technology called Docker that allows packaging up entire Linux software systems and easily running them elsewhere.

To replicate an individual simulation treatment with the single-market-robot-simulator software on Windows 10 Pro or Windows 10 For Education, follow these steps:

1. Find an appropriate computer.
  Consider potential for abusive users or intruders to use Docker Desktop to run unauthorized spy software or other unwanted software, access to sensitive information (documents; remote capture), and local rules about installing software.
  At least 2 CPU cores, 8 GB Ram, 20GB free disk space.
  A copy of the dataset should be downloaded and unzipped.
  Mac or Linux: see special section below for minor changes to these procedures
  Not compatible: Windows 10 for Home; older version of Windows; most early Chromebooks
2. Install Docker Desktop
  This is only required once per computer.
  Install Docker Desktop [9] by visiting the referenced URL, clicking the “Download Desktop” button, and running the associated installer. This is free open source software from the Docker company. Docker Desktop allows installing previously uploaded 3rd party software from Docker Hub [10] to run in an isolated copy of Linux running alongside Windows.
3. Open Command Prompt. Type “command prompt” in the search box at the lower left of Windows 10 to find the app that creates (black background) command windows.
4. docker run hello-world
  entered into the command window will test your docker installation and internet connection to Docker Hub. You should see some messages, including “Hello from Docker! This message shows that your installation appears to be working correctly.
  ” See also Docker's “Get Started” page [11].
5. mkdir C:∖work
  entered into the command window creates a folder for containing the input and output files. You can choose a different folder location - but then you must change subsequent steps to match.
6. cd C:∖work
  entered into the command window changes directory to C:∖work
  The prompt should change to indicate the command window is working with C:∖work. If there is an error, check step #5.
7. Go to the file manager and find the dataset folder and the sim.json file for the treatment you wish to replicate. Copy the sim.json file to C:∖work.
  This example uses the dataset file /primary/market1/data/10/sim.json
  (treatment variables: primary, market 1 values/costs, 50% SB robots)
8. type sim.json
  entered into the command window displays the sim.json file for confirmation.
  Note: this command is “type sim.json”, not merely the act of typing “sim.json” alone.
  If the sim.json file does not exist, check step #7.
  Confirm you can scroll the screen to see the appropriate buyerValues, sellerCosts, and robot types in the output. Optionally compare values and costs with the design specified in Ref. [1] for market 1.
9. docker run -it -v c:∖work:/work drpaulbrewer/single-market-robot-simulator:4.3.0
  entered into the command window runs the simulation treatment defined in C:∖work∖sim.json
  You may be asked to allow access to “C:∖work” by Docker Desktop. The simulator will not run properly if you refuse permission to read and write files in C:∖work.
  The simulation runs without any user interface or indication of progress. Depending on the speed of your computer, it could take anywhere from a few minutes to 30 minutes. If you get an immediate error, check the docker command carefully for minor errors.
  During the simulation, if you open another window and look in your work directory (C:∖work), you will find a number of.csv files have been created and are filling up with data. The file “period” contains the current period number. When it reaches 10000, the simulation will stop and the command window will provide a new input line.

Mac and Linux users (not Windows users) should modify the above procedure as follows:

• Step 1: Linux based Docker can be run on weaker systems (1 core; less than 2GB of RAM) because Docker will run natively on Linux.
• Step 2: Docker Desktop exists for Mac. On Linux, use a recent version of Docker CE.
• Step 3: Mac and Linux have a “terminal” app for command input.
• File locations are specified using “/” on Mac and Linux instead of “∖” for Windows files.
• Step 8: “more sim.json” is the command to view the file
• Step 9: Depending on installation, Mac or Linux users may require “root” or “sudo.” Docker can be set up to be accessible to ordinary users, but that is not always the case.
• Step 9: A more convenient specification of the “-v” option is available : "-v $PWD:/work" indicates that the current directory is to be linked to the /work container directory.

On completing the simulation, you should have a work directory with various.csv files containing market data.

As a next step in this replication, you may calculate the Gini coefficient from total profit in the simulation treatment aggregated over 10,000 periods.

The Gini calculation procedure involves entering the following docker command in a command window, from the work directory:

docker run -it -v c:∖work:/work drpaulbrewer/gini-csv gini-csv -n -s -m y -r 4 work/profit.csv

- This is a single long command. Check for errors before pressing enter.
- Check gini-csv is repeated twice, but in different contexts. This is necessary because docker must first fetch and use the drpaulbrewer/gini-csv container and then run the gini-csv command.
- Check that after “gini-csv” each of n, s, m, r has a dash in front. The y and 4 do not have a dash.

The output, the Gini coefficient for total profits, is sent to the command window.

This replication procedure has been tested on:

• Windows 10 Pro, Intel i7
• Mac Mini, Intel i5.
• Linux, using a remote virtual machine running on Free Google Cloud Shell [12].
• Linux, using a remote virtual machine running on Amazon AWS, type ‘t2.micro’ (also free)

Gini Coefficients obtained from these four replications ranged from 0.2368 to 0.2379.
